# Improvement of Low-Temperature Performance of Buton Rock Asphalt Composite Modified Asphalt by Adding Styrene-Butadiene Rubber

**DOI:** 10.3390/ma12152358

**Published:** 2019-07-24

**Authors:** Xiyan Fan, Weiwei Lu, Songtao Lv, Fangwei He

**Affiliations:** National Engineering Laboratory of Highway Maintenance Technology, Changsha University of Science & Technology, Changsha 410114, China

**Keywords:** road engineering, low-temperature performance, BRA-modified asphalt, BRA-SBR composite modified asphalt

## Abstract

To improve the low-temperature performance of the Buton rock asphalt (BRA)-modified asphalt, styrene-butadiene rubber (SBR) was added to it. The BRA-modified asphalt and SBR-BRA composite modified asphalt were prepared by high-speed shearing method. The penetration, softening point, ductility, and Brookfield viscosity of the two kinds of asphalt were measured. The dynamic shear rheometer (DSR) and the beam bending rheometer (BBR) were employed to research the performance of BRA-modified asphalt by adding SBR. The results showed that the pure asphalt in BRA was the main reason to reduce the low-temperature performance of neat asphalt when the content of BRA was 19%. However, the ash in BRA was the main factor to reduce the low-temperature performance when its content was more than 39.8%. When the BRA content was 59.8%, the SBR-BRA composite modified asphalt with SBR contents of 2%, 4%, 6%, and 8%, and it shows that the penetration and ductility of the BRA-modified asphalt are increased by the addition of SBR. The equivalent brittle point was reduced, the stiffness modulus was decreased, and the creep rate was increased. At the same time, the Brookfield viscosity was reduced and the rutting factor was increased. The stiffness modulus of the SBR-BRA composite modified asphalt mixture was increased. That is to say, when SBR was mixed into the BRA-modified asphalt, the low-temperature performance could be remarkably improved based on ensuring high-temperature performance. The low-temperature index of composite modified asphalt was analyzed. It was recommended to apply the equivalent brittle point to evaluate the low-temperature performance of SBR-BRA composite modified asphalt.

## 1. Introduction

By the end of 2018, the total mileage on China’s highways was 4.85 million kilometers, including 142,600 km of expressways. Most of the pavement structures are asphalt pavement [1,2,3,4,5]. However, with the growth of the traffic volume, neat asphalt binder is difficult to meet modern transport development demands and needs to be modified. Buton rock asphalt (BRA) is a kind of asphalt with excellent stability formed by the action of different natural environmental factors [6]. It has excellent compatibility with asphalt, low production cost, and convenient transportation [7]. Therefore, rock asphalt as a modifier to modify neat asphalt has prominent advantages [8,9]. Engineering practice shows that the application of rock asphalt-modified asphalt can improve the rutting and other diseases caused by an overload on the road surface, improve the service performance of the road surface, and extend the service life of it. It is also reported that the added BRA can enhance the skid resistance of the asphalt pavement [10]. It is indeed an excellent road asphalt modifier [11,12]. Other researches are shown that BRA-modified asphalt has excellent high-temperature stability and anti-aging performance, but its addition to neat asphalt destroys the low-temperature cracking resistance [13,14,15]. To prevent low-temperature shrinkage cracks and improve the service capacity of the highway, it is necessary to seek other kinds of modifiers to modify the neat asphalt [16], so that the composite-modified asphalt can improve the high-temperature performance as well as the low-temperature performance.

SBR is a modifier for polymer-modified asphalt. Because of its good compatibility with asphalt and its rich content of polycyclic aromatic hydrocarbons, it can significantly improve the low-temperature performance of neat asphalt. Besides, SBR modification technology is mature and has been widely used in asphalt modification [17,18,19]. The results have shown that SBR is a linear polymer material with high molecular weight (100,000–1.5 million). SBR will increase the average molecular weight of modified asphalt so that the modified asphalt forms a mosaic structure with a large surface area and has high surface energy. When the temperature decreases, SBR particles can play a role in toughening and plasticizing, offset part of the load effect, and hinder the further expansion of micro-cracks [20,21]. It has also been shown that SBR could reduce the hardening of asphalt during oxidative aging [22,23,24,25]. Therefore, the performance of SBR-modified asphalt is relatively good, and especially at a lower temperature, can show good flexibility, ductility, and crack resistance. Previous studies have shown that the comprehensive performance of BRA–SBR composite modified asphalt was better than styrene-butadiene-styrene (SBS)-modified asphalt and SBR-modified asphalt [26,27]. The study [28] has also shown that as the amount of BRA increases, the adhesion of the composite-modified asphalt to the aggregate and the Brookfield viscosity increased.

Although many studies have shown that the low-temperature performance of BRA-modified asphalt deteriorated, most of the studies have employed mechanism analysis [29,30]. It has not been found which component of BRA has a negative influence on the low-temperature performance of neat asphalt. Moreover, the studies have only shown that SBR could improve the low-temperature properties of neat asphalt [31], but its performance as a composite modifier currently lacks systematic research. Aiming at these issues, this study first characterizes the asphalt modified with BRA and BRA-ash, respectively. The performance between the BRA- and BRA-ash-modified asphalt binder was compared to determine whether the BRA-asphalt or BRA-ash content play a major role in asphalt modification. Then, based on the characterization results, the low-temperature performance of the BRA-modified asphalt was further improved with the added SBR content. The sensitivity of the low-temperature index to the content of SBR was analyzed.

## 2. Materials Preparation and Test Method

### 2.1. Materials Preparation

The material used in this paper is AH-70# neat asphalt, produced from Indonesia’s Buton rock asphalt, SBR latex. Technical indicators are shown in Table 1, Table 2 and Table 3. The results show that the technical indicators of all raw materials are in line with the norms.

**Figure 1:**
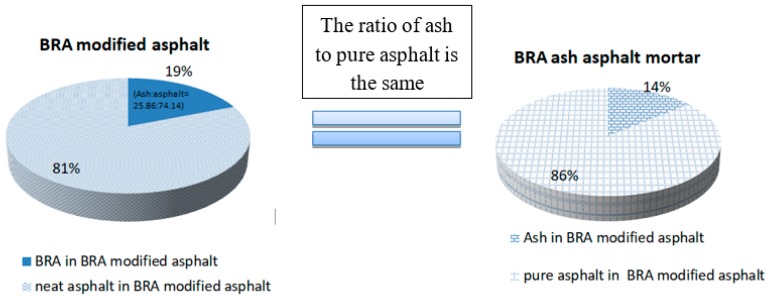
The ratio of ash to pure asphalt is the same.

Based on the research of other scholars [32,33,34], the external blending method was used to determine that the blending amount of BRA was 19%, 39%, 58%, 77%, and 97%. This reflects the mass ratio of BRA to modified asphalt. According to the principle that the ratio of ash to pure asphalt is the same, the amount of Buton rock ash mortar is shown in Table 4. For example, when the amount of BRA is 19%, pure asphalt accounts for 5% and ash accounts for 14% in the BRA-modified asphalt. It means that the percentage of pure asphalt is the same in BRA-modified asphalt and BRA ash mortar. The percentage of ash is the same in BRA-modified asphalt and BRA ash mortar, as shown in Figure 1. The amount of SBR is also determined according to the research of other scholars [35].

Studies [36] have shown that when the BRA dosage range is between 40% and 80% (mass ratio), the BRA-modified asphalt has the same rutting resistance as the SBS-modified asphalt and has excellent high-temperature performance. However, according to the BBR test data of BRA-modified asphalt in this paper, 58% of the amount of BRA-modified asphalt reached the limit of low-temperature performance of asphalt at −6 °C. BRA content of 58% of was selected to prepare BRA-SBR composite modified asphalt.

**Figure 2:**
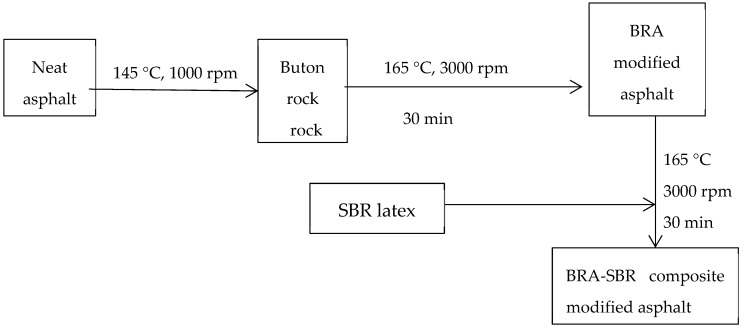
Flow chart for preparing modified asphalt.

The high-speed shear and induction cooker were used to heat the AH-70# neat asphalt binder to 140–145 °C before mixing of modified asphalt binder. The BRA with different proportions was added to the asphalt binder. The high-speed shear was turned for 30 min to mix the BRA and neat asphalt when the temperature was controlled at 165 °C, and then different amounts of SBR were added. The BRA-SBR composite modified asphalt was prepared [37]. The flow chart is shown in Figure 2.

The Buton rock asphalt ash is obtained by burning the Buton rock asphalt at a high temperature. Its main component is CaCO_3_, and its decomposition temperature is 825–896.6 °C, and the melting point is 1339 °C. To ensure that the microstructure of the Buton rock asphalt is not resolved, the muffle furnace’s combustion temperature is set to 482 °C to burn the BRA. The BRA ash mortar is prepared in a similar manner to the BRA-modified asphalt.

### 2.2. Test Method

The penetration test is a commonly used method for determining the consistency of asphalt. Penetration tests of 3 temperatures were carried out, and the penetration index (PI) and the equivalent brittle point (T_1.2_) were calculated. The penetration index is an indicator of the temperature sensitivity of asphalt. T_1.2_ means the corresponding temperature at which the penetration of the asphalt is 1.2. It reflects the low-temperature properties of asphalt.

The ductility test was carried out at 10 °C. The relation curve of load and ductility of asphalt can be obtained from the force ductility test, and the area enclosed by the curve and X-axis is usually called the abruption power. The abruption power (A) represents the work required by the external force in the process of stretching to the breaking of asphalt. This index takes into account the deformation and tension in the whole test process, and can better evaluate the viscosity and toughness of asphalt at low-temperature compared with the ductility. The ratio of the ductility to the tension is taken as the compliance in extension, and the value is used to measure the low-temperature viscosity and toughness of asphalt, which can better reflect the low-temperature performance of asphalt [38]. 

The low-temperature bending beam rheology (BBR) test was specified by Superpave as a test to evaluate the low-temperature properties of asphalt. Strategic Highway Research Program (SHRP) believes that the cracking of the road surface is related to the stiffness of the asphalt mixture at 7200 s. If it is less than or equal to 200 MPa, the cracking is small. It is difficult to control the temperature to be stable when the loading time is 7200 s. According to the time–temperature equivalent principle, the creep stiffness of 7200 s is equivalent to the test result of the BBR test for 60 s. The test results of BBR are expressed as creep stiffness and creep rate at 60 s.

In order to further observe the improvement of the low-temperature performance of BRA-modified asphalt by SBR, the low-temperature creep bending test was carried out in this paper. The composite modified asphalt was prepared by a BRA content of 58% and an SBR content of 5%. The failure strain index of the low-temperature bending test was used to evaluate the low-temperature performance of the modified asphalt mixture. The bending strength and the bending strain at the time of fracture of the test piece are used to calculate the stiffness modulus at the time of failure of the test piece [39]. The test conditions are shown in Table 5.

To make the study more complete, the high-temperature performance of the BRA-SBR composite modified asphalt was also observed through the Brookfield rotary viscosity test and the DSR (dynamic shear rheometer) test. The Brookfield viscosity test temperatures were 135 °C, 155 °C, and 175 °C. The speed is set to 10 r/min. The initial recording temperature of the asphalt dynamic shear rheological test is 46 °C, which is recorded every 6 °C to obtain the complex modulus, phase angle, and rutting factor of the modified asphalt.

## 3. Test Result

### 3.1. Penetration Test

The penetration at 15 °C was related to the low-temperature performance of asphalt. The higher the penetration at 15 °C, the better the low-temperature performance of asphalt [40]. Table 6 can conclude that when BRA content was 19%, 39%, 58%, 77%, and 97%, compared with the neat asphalt, the penetration of BRA-modified asphalt (15 °C) was reduced by 18.3%, 25.5%, 37.8%, 45.4%, and 55.4%. As the temperature increased, the penetration increased. However, as the BRA dosage increases, the amplitude decreases. BRA is a granule. Its addition can reduce the rheological properties of asphalt. At the same time, the equivalent brittle point T_1.2_ was increased by 0.75 °C, 1.51 °C, 2.93 °C, 3.61 °C, and 4.42 °C. It indicates that the low-temperature crack resistance of BRA-modified asphalt decreases, that is, the hardness of BRA-modified asphalt increases at a low temperature, and brittle fracture is likely to occur when the BRA-modified asphalt is stressed at a low temperature [41]. As the content of BRA increased from 0% to 97%, the penetration index of BRA-modified asphalt increased from −0.602 to 0.346, indicating that the thermal sensitivity of BRA-modified asphalt was improved.

Table 7 can conclude that when the ash content of BRA was 14%, 29%, 43%,57%, and 72%, the penetration (15 °C) of the BRA ash asphalt mortar was reduced by 4%, 12%, 20%, 28%, and 36%; moreover, the equivalent brittle point T_1.2_ increased by 0.21 °C, 0.92 °C, 1.26 °C, 1.85 °C, and 2.33 °C compared to the neat asphalt. It is shown that with the increase of BRA ash content, the hardness of BRA ash asphalt mortar increased at low temperatures, and the low-temperature crack resistance decreased. As the BRA ash content increased from 0% to 72%, the penetration index of BRA ash asphalt mortar increased from −0.662 to −0.174, indicating that the temperature sensitivity of the BRA ash asphalt mortar was improved.

From Table 8 and Table 9, it can be found that the addition of BRA and BRA ash reduced the penetration of neat asphalt. When the amount was small, pure asphalt in BRA played a major role. When the amount was high, ash in BRA was the main one. It can be seen from Table 9 that the addition of BRA and BRA ash increased the equivalent brittle point of the neat asphalt. When the dosage was low, pure asphalt in BRA played a significant role. When the dosage was high, ash in BRA played the main character. That is, as the amount of BRA is increased, the weakening effect of ash in BRA on the low-temperature performance of neat asphalt is more pronounced.

As can be seen from Table 10, with the increase of SBR, the penetration and penetration index of BRA-SBR composite modified asphalt increased. The greater the penetration, the softer the asphalt. The greater the penetration index, the lower the temperature sensitivity of the asphalt. It reflects that the addition of SBR improved the low-temperature performance of the BRA-SBR composite modified asphalt. When the SBR content was 2%, 4%, 5%, 6%, and 8%, the equivalent brittle point of the BRA-SBR composite modified asphalt as 2.42 °C, 3.19 °C, 4.35 °C, 5.96 °C, and 7.99 °C lower than that of the BRA-modified asphalt. The equivalent brittle point (T_1.2_) is a low-temperature indicator. The smaller it is, the better the low temperature crack resistance of the composite-modified asphalt. This shows that the BRA–SBR composite modified asphalt has the best low-temperature performance, followed by the neat asphalt and the BRA-modified asphalt. With the increase of SBR, the equivalent brittle point of BRA-SBR composite modified asphalt increased first and then decreased.

### 3.2. Force Ductility Test

**Figure 3:**
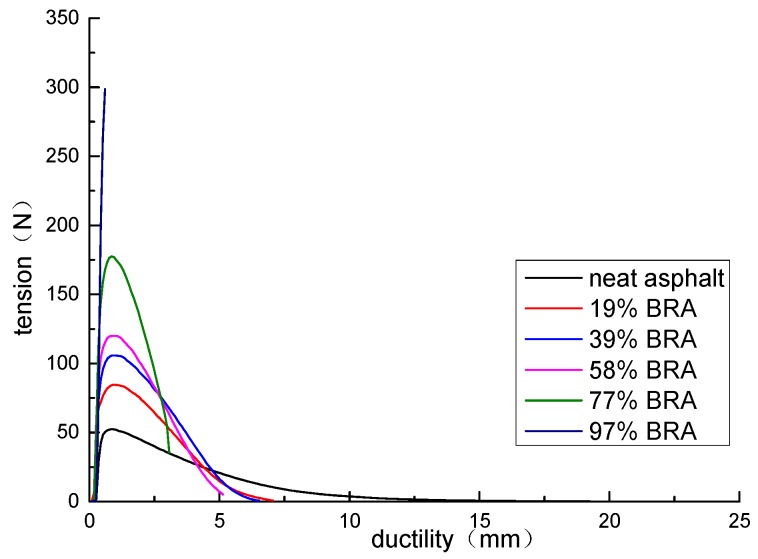
Relationship between tensile force and ductility of BRA-modified asphalt.

It can be seen from Table 11 and Figure 3 that the 10 °C ductility of the BRA-modified asphalt gradually decreased as the amount of BRA increased. After blending 19% and 39% of BRA, the variation of BRA-modified asphalt and neat asphalt is the same. The tensile force increased first and then gradually decreased to zero. The specimen did not suddenly break, which indicates that the material also has toughness and tenacity. However, the peak tensile strength of BRA-modified asphalt increased, indicating that the viscoelasticity of BRA-modified asphalt increased. When 58%, 77%, and 97% BRA were added, the tensile force of BRA-modified asphalt changed abruptly. Especially when 97% BRA was added, the tensile force reached its peak value and breaks suddenly. Compliance in extension is an elastic constant equal to the ratio of strain to stress. The greater the compliance, the easier it is to deform. As shown in Table 11, the tensile compliance decreased as the BRA content increased. It shows that the low-temperature performance of BRA-modified asphalt was reduced. The larger power was correlated to the higher the energy absorption during stretching. That is, asphalt had good toughness and strong fatigue resistance. As the content of BRA increased, the power decreased and the toughness deteriorated.

**Figure 4:**
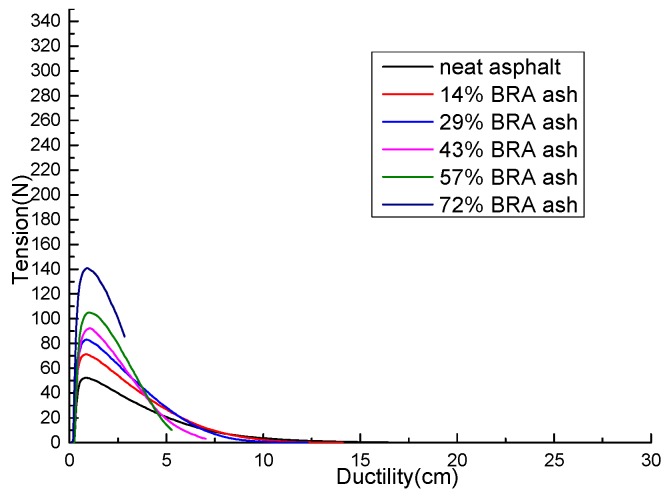
Comparison between tensile force and ductility of asphalt mortar with different BRA ash content.

The curve of Figure 4 can be seen as a stress-strain curve, and as the ductility increased, the force also increased. When the peak was reached, the ductility continued to increase and the force began to decrease. That is, the first was the elastic deformation process, followed by the plastic deformation process. It can be seen from Table 12 and Figure 4 that the 10 °C ductility of the BRA ash asphalt mortar also exhibited a gradually decreasing change as the BRA content increased. When the ash content of BRA was 14% and 29%, the variation of BRA ash asphalt mortar was consistent with that of neat asphalt. The tensile force decreased gradually to zero with the increase of ductility, which indicates that the material has plastic characteristics. After the incorporation of 43%, 57%, and 72% BRA ash, the curve of the BRA ash asphalt mortar did not decay to 0, which indicates that the material has brittleness characteristics. With the addition of BRA ash, the compliance and power of BRA ash asphalt mortar were also reduced. The reason for the smaller amplitude than BRA-modified asphalt is due to the pure asphalt in BRA. It can improve the rheological properties of asphalt, making the asphalt softer, resulting in poor asphalt low-temperature performance.

Based on the above analysis, the BRA-modified asphalt contains a large number of irregular BRA particles, which quickly causes stress concentration inside the modified asphalt. As shown in Table 13, when the ductility test was carried out, BRA caused the modified asphalt to decrease in ductility, which is the central role of BRA ash. Besides, the pure asphalt in BRA improved the cohesive properties of the asphalt, resulting in an increase in the tensile strength of the BRA-modified asphalt and a decrease in flexibility. Under the combined effect of these two factors, the low-temperature performance of BRA-modified asphalt was inferior to that of neat asphalt.

It can be seen from Table 14 that when SBR output was 2%, 4%, 5%, 6%, and 8%, the ductility increased by 4.59 cm, 8.04 cm, 11.73 cm, 14.31 cm, and 15.47 cm, respectively. The low-temperature performance of asphalt was effectively improved by adding SBR, and the effect was better with the increase of SBR content. With the addition of SBR, the compliance and power of BRA-SBR composite modified asphalt increased. It indicates that the SBR improve the toughness and fatigue resistance of BRA-modified asphalt.

**Figure 5:**
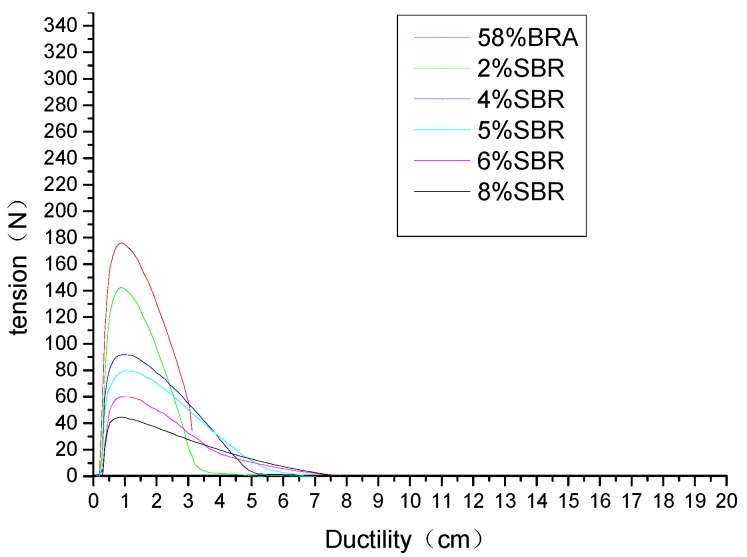
Ductility test of BRA–SBR composite modified asphalt.

The ductility test showed that the addition of BRA reduced penetration, compliance, and power. That is, the toughness and deformation ability of the asphalt deteriorated, which resulted in the poor low-temperature performance of the BRA-modified asphalt. Compared with BRA ash asphalt mortar, the magnitude of the deterioration was small, indicating that the presence of pure asphalt tends to cause poor low-temperature performance. SBR is an unsaturated olefin polymer that allows the asphalt to form a more stable colloidal structure. Therefore, the low-temperature performance is improved, as shown in Figure 5.

### 3.3. Low-Temperature Bending Beam Rheological Test

Analysis of Table 15 and Table 16 can be obtained:

At the same temperature, with the rise of BRA content, the stiffness modulus of BRA-modified asphalt increased, and the creep rate decreased. This shows that under constant load, the deformation of BRA-modified asphalt at the same temperature decreased with the increase of BRA content, and the stress relaxation performance of the material decreased and the low-temperature flexibility decreased.

BRA-modified asphalt could not meet the low-temperature performance requirement of −6 °C after the content of BRA was more than 58%, that is, the ash content was more than 39%. However, in the BRA ash asphalt mortar, the ash content greater than 43% met the low-temperature performance requirements of −6 °C. When the blending amount of BRA exceeded 19%, that is, the ash content was more than 14%, the BRA-modified asphalt could not meet the low-temperature performance requirement of −12 °C. In contrast, when the ash content was greater than 14% in the BRA ash asphalt mortar, the low-temperature performance requirement of −12 °C was satisfied. The reason is that when the blending amount of BRA is low, pure asphalt plays a major role in the modified asphalt, and when the amount of BRA is high, BRA ash plays a major role, as shown in Table 17 and Table 18.

It can be seen in Table 19 that when the temperature was −6 °C, the stiffness modulus of the BRA-SBR composite modified asphalt with the SBR parameter of 0% was 1.16 times the parameter of 2%. The creep rate of the composite-modified asphalt with the SBR parameter of 0% was 1.09 times the parameter of 2%. When the temperature was −12 °C, the stiffness modulus of the composite-modified asphalt with the SBR parameter of 0% was 1.18 times the parameter of 2%. The creep rate of the composite-modified asphalt with the SBR parameter of 0% was 1.05 times the parameter of 2%. The creep rate of BRA-SBR composite modified asphalt increased with the increase of SBR content, which indicates that SBR can improve the flexibility of BRA-SBR composite modified asphalt. When the temperature decreased, the effect of SBR improved the low-temperature performance of BRA-SBR composite modified asphalt more obviously.

As the SBR latex increased, the stiffness modulus decreased. That is, the deformation ability of the asphalt at a low temperature increased. The stress caused by the shrinkage strain of the asphalt was small, and the low-temperature crack resistance was excellent. As the SBR content increased, the creep rate increased. This shows that the flexibility of the composite-modified asphalt increased and it was not easy to crack. When the SBR parameter was 0, the stiffness modulus was the largest, and the 43% BRA ash asphalt mortar was the second. The neat asphalt was the smallest. It shows that the addition of BRA caused the low-temperature performance of the neat asphalt to deteriorate, and the addition of SBR improved the low-temperature performance of BRA-modified asphalt.

### 3.4. The Evaluation Index of BRA-SBR Composite Modified Asphalt under Low-Temperature

In this paper, the low-temperature performance of BRA-SBR composite modified asphalt was evaluated by penetration at 15 °C, equivalent brittle point, ductility at 10 °C, and stiffness modulus. Which index is more suitable to characterize the low-temperature performance of BRA-SBR composite modified asphalt is further studied.

The above studies show that penetration, ductility, creep rate, and equivalent brittleness point can reflect the low-temperature properties of asphalt. As the penetration increased, the asphalt became soft and the low-temperature performance was improved. As the ductility increased, the plastic deformation of the asphalt increased. The greater the creep rate, the stronger the low-temperature deformation ability of the asphalt and the better the low-temperature crack resistance of asphalt.

**Figure 6:**
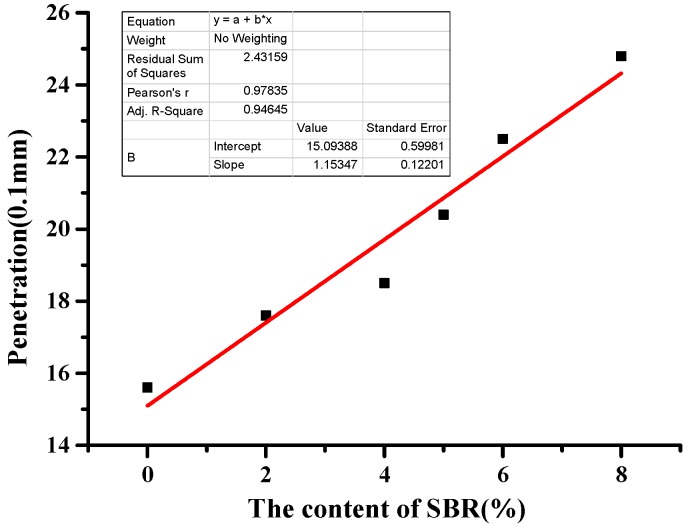
Fitting curves of SBR content and penetration.

**Figure 7:**
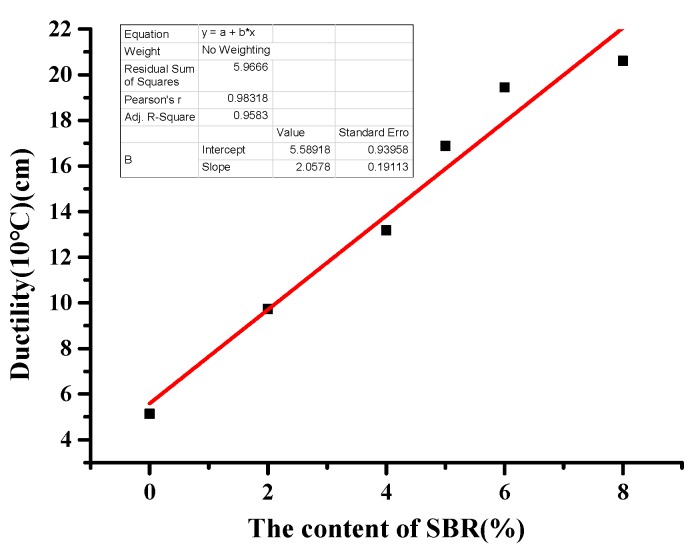
Fitting curves of SBR content and ductility.

**Figure 8:**
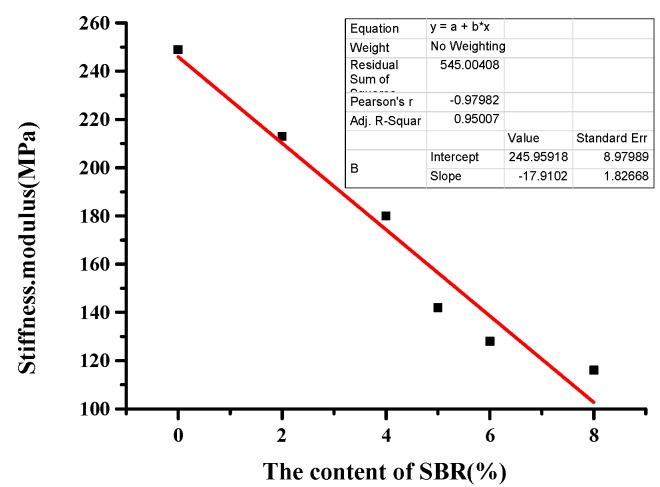
Fitting curves of SBR content and stiffness modulus.

**Figure 9:**
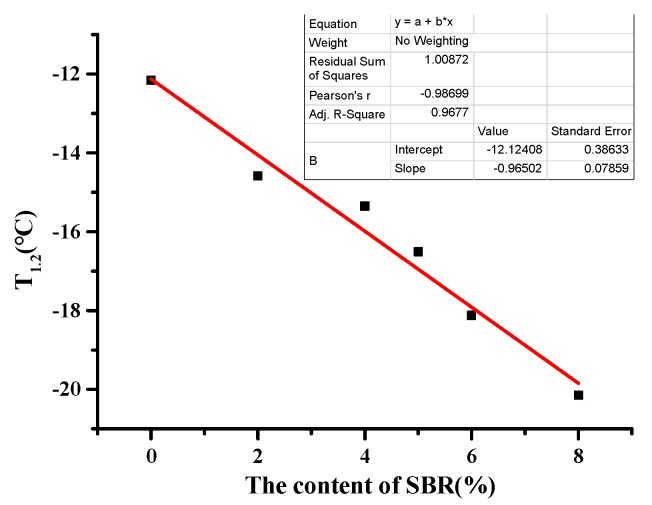
Fitting curves of SBR content and equivalent brittle point.

This paper fits the indicators of low-temperature index and SBR content. In order to analyze which indicator is more sensitive to low temperature performance, sensitivity was analyzed based on the slope. The greater the slope, the more sensitive it is to low-temperature performance. As can be seen from Figure 6, Figure 7, Figure 8 and Figure 9, the slope of Figure 7 is the largest. With the increase of SBR content, the changing trend of the ductility is the most sensitive. It can be concluded that ductility is the most suitable for evaluating the performance at a low temperature.

### 3.5. Low-Temperature Creep Bending Test of BRA-SBR Composite Modified Asphalt Mixture

**Figure 10:**
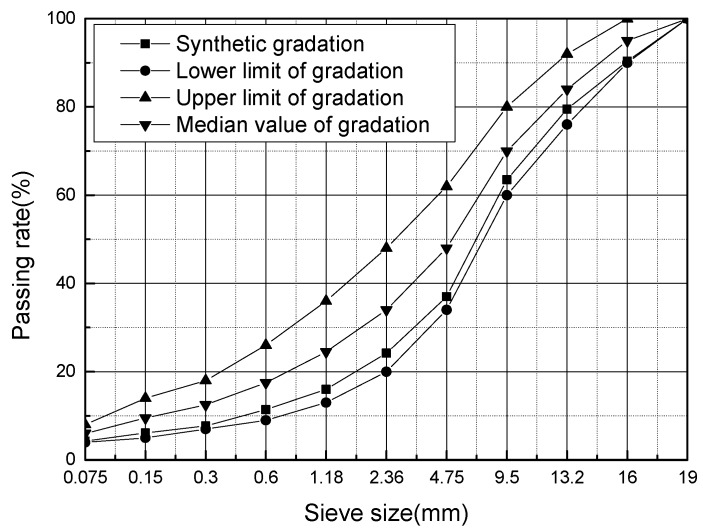
The aggregate gradation of AC-16C.

The performance indexes of asphalt are shown in Table 20. The dense skeleton type gradation of aggregates was chosen according to the “Specifications for Design of Highway Asphalt Pavement” (Figure 10) [42]. The optimum asphalt ratio was determined using Marshall Tests (Table 21).

As shown in Table 22: Among the three asphalt mixtures, BRA-SBR composite modified asphalt mixture had the highest flexural-tensile failure strength, followed by BRA-modified asphalt mixture and neat asphalt mixture. SBR can improve the ability of BRA-modified asphalt to withstand damage at a low temperature.

*The Technical Specification for Construction of Highway Asphalt Pavement* (ITGF 40-2004) takes the maximum tensile strain of beams in low-temperature bending test of asphalt mixture as the evaluation index of low-temperature tensile performance of asphalt mixture. The maximum tensile strain of ordinary asphalt mixture was more than 2000 when it was fractured, while that of modified asphalt mixture was more than 2500 when it was fractured. Among the three asphalt mixtures, only BRA-modified asphalt failed to meet the requirements of specifications. This is mainly due to the addition of Buton rock asphalt, which makes the flexural strain smaller, the overall brittleness of asphalt mixtures, and slightly decreases the crack resistance of asphalt mixtures. The low-temperature failure strain of BRA-SBR composite modified asphalt mixture was significantly improved, which indicates that its low-temperature performance was significantly improved.

In terms of stiffness modulus, the stiffness modulus of neat asphalt mixture was the lowest among the three asphalt mixtures, followed by BRA-SBR composite modified asphalt mixture, and BRA-modified asphalt mixture was the highest. This shows that BRA-modified asphalt caused the stiffness modulus of asphalt mixture to increase significantly, indicating that BRA weakens the low-temperature performance of asphalt mixture, and SBR improves the low-temperature performance of BRA-modified asphalt mixture.

### 3.6. High-Temperature Performance of BRA-SBR Composite Modified Asphalt

The test results from Table 23 show that the incorporation of SBR can reduce the Brookfield rotary viscosity. Moreover, when the SBR content was more than 5%, the Brookfield rotary viscosity of the BRA-SBR composite modified asphalt was smaller than that of the neat asphalt. That is, the workability of the BRA-SBR composite modified asphalt gradually became better as the amount of the SBR increased.

**Figure 11:**
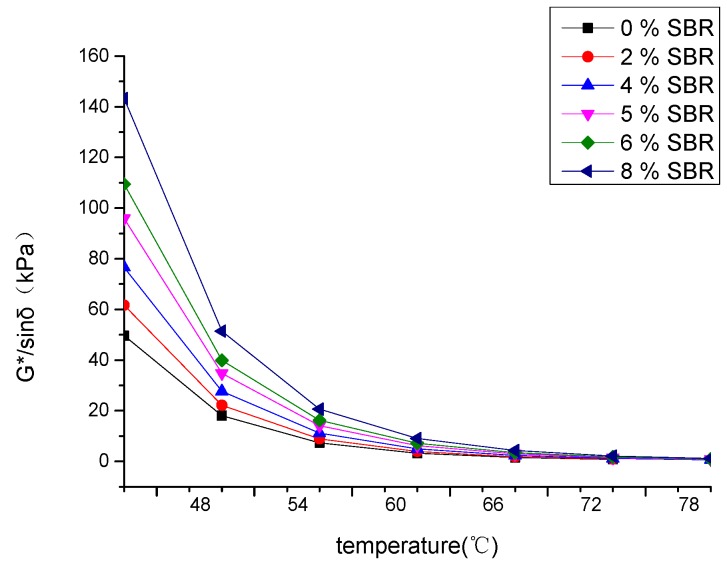
Rutting factor G*/sinδ with different temperatures.

It can be seen from Figure 11 that the Rutting factor decreased with increasing temperature. The rutting factors of BRA-SBR composite modified asphalt are larger than BRA-modified asphalt. It means that the BRA-SBR composite modified asphalt is more elastic. When the content of SBR increased, the elastic of BRA-SBR composite modified asphalt increased. It is potentially because the high temperatures of BRA-SBR composite modified asphalt was improved by SBR additives.

**Figure 12:**
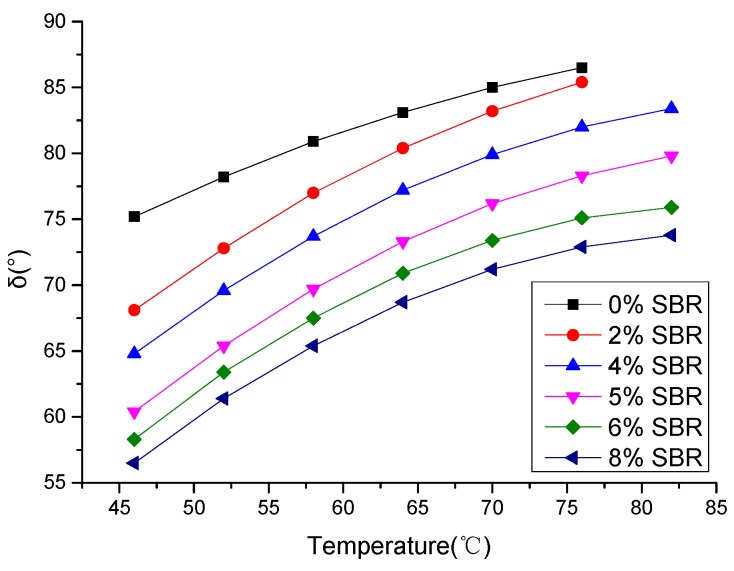
Phase angle δ with different temperatures.

It can be seen from Figure 12 that the phase angles δ of asphalt increased as the temperature increased. At the same temperature, the phase angle δ of BRA-SBR composite modified asphalt became smaller as the content of SBR increases. The phase angle δ of the BRA-SBR composite was smaller than BRA-modified asphalt, which means that the BRA-SBR composite modified asphalt is more elastic. When the content of SBR increased, the elasticity of BRA-SBR composite modified asphalt increased. There is potential that the high temperatures of asphalt binders and mixtures improved by SBR additives. The resistance of asphalt binders and mixtures to deformation is enhanced with improved durability.

In summary, when SBR is incorporated in the BRA-modified asphalt, the low-temperature performance can be remarkably improved on the basis of ensuring high-temperature performance. The reason is that SBR is an unsaturated olefin polymer, which can be dissolved in most of the solubility parameters and in the hydrocarbon solution close to styrene-butadiene rubber and the glass transition temperature is as low as −50 °C. BRA particles and neat asphalt have excellent compatibility. BRA particles can improve the poor compatibility of SBR with neat asphalt, enabling SBR and BRA particles as well as neat asphalt to form a more stable colloidal structure. When subjected to loads, micro-cracks appear, and SBR particles can play the role of toughening and plasticizing, offset some of the load effects, and hinder the further expansion of micro-cracks. Therefore, SBR can improve the low-temperature performance of BRA-modified asphalt, so that it can exhibit good flexibility, ductility, and crack resistance at lower temperatures.

## 4. Conclusions

This study aims to fill the knowledge gap on the effect of individual BRA component on the modification of asphalt binder and improve its low-temperature performance based on the obtained correlation. The performance difference between the BRA and BRA-ash modified asphalt was compared to determine the influence of a single component. Then, the SBR content was further applied to improve low-temperature performance of the BRA-modified asphalt. The main conclusion of this study was shown below.

1. The individual modification effect of BRA-binder and BRA-ash content was determined based on the characterization on BRA and BRA-ash modified asphalt, respectively. It was found that the asphalt was mainly affect by the BRA-binder content with a relatively low replacement ratio (within 20%), and the BRA-ash content played a main role in asphalt modification when the replacement ratio was relatively high (larger than 30%).
2. The addition of SBR can improve the low-temperature performance of BRA-modified asphalt. The ultimate failure strain and the failure strength were both enhanced with the added SBR content.
3. The correlation analysis indicated the ductility is more sensitive to the SBR content and hence, the test was recommended to evaluate the low-temperature performance of SBR-modified asphalt.

## Figures and Tables

**Table 1:** Technical properties of 70# neat asphalt.

Technical Indicators	Industry Standard	Test Results
Penetration (25 °C,100 g, 5 s) (0.1 mm)	60–80	68.2
Ductility (5 cm/min, 15 °C) (cm)	≥100	>100
Softening point (°C)	≥46	49.1
Density (15 °C) (g/cm^−3^)	-	1.03

**Table 2:** Main technical indexes of Buton rock asphalt.

Technical Indicators		Industry Standard	Test Results
Appearance		Brown Powder	Brown Powder
Ash content (%)		<75	74.14
Solubility (%)		>25	25.86
Water content (%)		<2	0.97
Particle size range (%)	4.75 mm	100	100
	2.36 mm	90–100	100
	0.6 mm	10–60	100

**Table 3:** Technical performance of styrene-butadiene rubber (SBR) latex.

Property	Test Result	Specification of Experimental Methods
Appearance	White Latex	-
Molecular weight	50,000	GB/T12005.10-1992
Mooney viscosity (mPa·s)	4000	GB/T1231.1

**Table 4:** Buton rock asphalt (BRA) and BRA ash content comparison table.

BRA Content	BRA Ash Content
0.19	0.14
0.39	0.29
0.58	0.43
0.77	0.57
0.97	0.72

**Table 5:** Test conditions of low-temperature creep bending test.

**Test Conditions**	**Specimen Size**	**Temperature**	**Loading Frequency**
	250 mm × 30 mm × 35 mm	−10 °C	50 mm/min

**Table 6:** Test results of BRA-modified asphalt.

Property		Unit	BRA (%)					
			0	19	39	58	77	97
Penetration	15 °C	0.1 mm	25.1	20.5	18.7	15.6	13.7	11.2
	25 °C		68.2	53.7	46.9	39.0	33.9	28.2
	30 °C		114.4	87.5	79.4	64.5	54.5	41.1
PI			−0.602	−0.321	−0.258	−0.149	0.019	0.346
T_1.2_		°C	−15.09	−14.34	−13.58	−12.16	−11.48	−10.67

**Table 7:** Penetration test results of the BRA ash asphalt mortar.

Property		Unit	The Content of BRA Ash (%)					
			0	14	29	43	57	72
Penetration	15 °C	0.1 mm	25.1	23.9	21.7	19.8	18.2	16.8
	25 °C		68.2	63.9	58.2	51.5	46.8	40.8
	30 °C		114.4	107.5	96.2	85.3	77.3	70.4
PI			−0.602	−0.539	−0.488	−0.35	−0.284	−0.174
T_1.2_		°C	−15.09	−14.88	−14.17	−13.83	−13.24	−12.76

**Table 8:** Percentage of penetration of different components in BRA.

BRA Content (%)	BRA Ash Content (%)	Penetration (15 °C)		The Proportion of Ash and Pure Asphalt in the Difference between the Penetration of BRA Modified Asphalt and Neat Asphalt	
		BRA Modified Asphalt	BRA Ash Asphalt Mortar	Ash (%)	Pure Asphalt (%)
0	0	25.1	25.1	0	0
19	14	20.5	22.5	57	43
39	29	18.7	21.7	53	47
58	43	15.6	19.8	56	44
77	57	13.7	18.2	61	39
97	72	11.2	16.8	60	40

**Table 9:** Percentage of equivalent brittle point of different components in BRA.

BRA Content (%)	BRA Ash Content (%)	T_1.2_ (°C)		The Proportion of Ash and Pure Asphalt in the Difference between the Equivalent Brittle Point of BRA Modified Asphalt and Neat Asphalt	
		BRA Modified Asphalt	BRA Ash Asphalt Mortar	Ash (%)	Pure Asphalt (%)
0	0	−15.09	−15.09	0	0
19	14	−14.34	−14.88	28	72
39	29	−13.58	−14.57	66	34
58	43	−12.16	−13.83	43	57
77	57	−11.48	−13.64	40	60
97	72	−10.67	−12.98	48	52

**Table 10:** Penetration of BRA-SBR composite modified asphalt.

Property		Unit	The Content of SBR (%) (The Content of BRA is 58%)					
			0	2	4	5	6	8
Penetration	15 °C	0.1 mm	15.6	17.6	18.5	20.4	22.5	24.8
	25 °C		39	41.4	43.4	48	57.5	63.8
	30 °C		64.5	69.1	71.9	79	84.3	89.3
PI			−0.149	0.127	0.173	0.185	0.239	0.406
T_1.2_		°C	−12.16	−14.58	−15.35	−16.51	−18.12	−20.15

**Table 11:** Force ductility test result of BRA-modified asphalt.

BRA Content (%)	Ductility (cm)	F_Max_ (N)	Compliance in Extension	A (J)
0	24.34	52.48	0.464	1265.708
19	7.05	84.56	0.083	269.8131
39	6.51	105.74	0.062	247.9926
58	5.14	119.96	0.043	170.0564
77	3.07	177.5	0.017	73.96904
97	0.60	298.56	0.002	1.63564

**Table 12:** Force ductility test result of BRA ash asphalt mortar.

BRA Ash Content (%)	Ductility (cm)	F_Max_ (N)	Compliance in Extension	A (J)
0	24.34	52.48	0.464	1265.708
14	14.09	71.36	0.197	685.8082
29	12.28	83.3	0.147	553.6637
43	7.02	92.42	0.076	263.8006
57	5.28	105.12	0.05	185.625
72	2.85	140.8	0.02	73.196

**Table 13:** Percentage of ductility of different components in BRA.

BRA Content (%)	BRA Ash Content (%)	Ductility (cm)		The Proportion of Ash and Pure Asphalt in the Difference between the Ductility of BRA Modified Asphalt and Neat Asphalt	
		BRA Modified Asphalt	BRA Ash Asphalt Mortar	Ash (%)	Pure Asphalt (%)
0	0	24.34	24.34	0	0
19	14	7.05	14.09	59	41
39	29	6.51	12.28	68	32
58	43	5.14	7.02	90	10
77	57	3.07	5.28	90	10
97	72	0.60	2.85	91	9

**Table 14:** Results of 10 °C ductility test of BRA-SBR composite modified asphalt.

SBR Content (%)	Ductility (cm)	F_Max_ (N)	Compliance in Extension	A (J)
0	5.14	119.96	0.043	170.0564
2	9.73	97.48	0.100	212.8536
4	13.18	84.98	0.155	258.7621
5	16.87	76.35	0.221	325.4685
6	19.45	65.12	0.299	512.5346
8	20.61	58.32	0.353	623.851

**Table 15:** Beam bending rheometer (BBR) test results of BRA-modified asphalt.

T (°C)	Test Results	Amount of BRA (%)					
		0	19	39	58	77	97
−6	S (MPa)	74.9	115	186	249	328	437
	m	0.497	0.439	0.346	0.301	0.245	0.215
−12	S (MPa)	161	270	406	522	663	885
	m	0.427	0.379	0.284	0.253	0.215	0.204

**Table 16:** BBR test results of BRA ash asphalt mortar.

T (°C)	Test Results	Amount of BRA Ash (%)					
		0	14	29	43	57	72
−6	S (MPa)	74.9	94.2	141	179	208	264
	m	0.497	0.461	0.388	0.346	0.340	0.316
−12	S (MPa)	161	208	266	347	485	637
	m	0.427	0.404	0.356	0.306	0.278	0.258

**Table 17:** Percentage of stiffness modulus of different components in BRA at −6 °C.

BRA Content (%)	BRA Ash Content (%)	Creep Rate		The Proportion of Ash and Pure Asphalt in the Difference between the Stiffness Modulus of BRA Modified Asphalt and Neat Asphalt	
		BRA Modified Asphalt	BRA Ash Asphalt Mortar	Ash (%)	Pure Asphalt (%)
0	0	0.497	0.497	0	0
19	14	0.439	0.461	62	38
39	29	0.346	0.388	72	28
58	43	0.301	0.346	77	23
77	57	0.245	0.340	62	38
97	72	0.215	0.316	64	36

**Table 18:** Percentage of stiffness modulus of different components in BRA at −12 °C.

BRA Content (%)	BRA Ash Content (%)	Creep Rate		The Proportion of Ash and Pure Asphalt in the Difference between the Stiffness Modulus of BRA Modified Asphalt and Neat Asphalt	
		BRA Modified Asphalt	BRA Ash Asphalt Mortar	Ash (%)	Pure Asphalt (%)
0	0	0.427	0.427	0	0
19	14	0.329	0.404	23	77
39	29	0.284	0.356	50	50
58	43	0.253	0.306	70	30
77	57	0.215	0.278	70	30
97	72	0.204	0.258	76	23

**Table 19:** BBR test results of BRA-SBR compound modified asphalt.

Amount of SBR (%)		0	2	4	5	6	8
*S* (MPa)	−6 °C	249	213	180	142	128	116
	−12 °C	522	436	376	297	265	247
*m*	−6 °C	0.301	0.327	0.344	0.367	0.388	0.409
	−12 °C	0.253	0.265	0.290	0.327	0.333	0.346
*m*/*S* (MP^−1^)	−6	0.001209	0.001535	0.001911	0.002585	0.003031	0.003526
	−12	0.000485	0.000608	0.000771	0.001101	0.001257	0.001401

**Table 20:** Performance Index of BRA–SBR composite modified asphalt.

Type of Asphalt	Penetration 25 °C, 100 g, 5 s (0.1 mm)	Softening Point TR&B (°C)	Ductility 10 °C (cm)	Relative Density
neat asphalt	68.2	49.1	24.34	1.029
BRA modified asphalt	39.0	61.4	5.14	1.045
BRA-SBR compound modified asphalt	48.0	63.2	16.87	1.036

**Table 21:** Marshall test results at optimal asphalt content.

	Optimal Asphalt Content (%)	Bulk Specific Gravity (g·cm^−3^)	The Volume of Air Voids VV (%)	Voids Filled with Asphalt VFA (%)	Voids in Mineral Aggregate VMA/%	Marshall Stability (kN)	Flow Value (mm)
neat asphalt mixture	5.2	2.469	4.3	69.3	14	16.21	3.2
BRA modified asphalt mixture	4.7%	7.8	15.0	45.1	16.26	2.8	2.536
BRA-SBR compound modified asphalt mixture	4.7%	6.5	13.7	52.8	16.63	3.1	2.401

The low-temperature creep bending test results are as follows.

**Table 22:** Creep bending test results of BRA–SBR compound modified asphalt mixture.

Property	Specimen	Flexural Tensile Strength (MPa)	Average Value (MPa)	Failure Strain (με)	Average Value (με)	Stiffness Modulus (MPa)
neat asphalt mixture	1	7.12	7.27	2158	2164.3	3823.8
	2	7.17		2238		
	3	7.51		2097		
BRA modified asphalt	1	8.91	8.75	1544	1484.0	5898.5
	2	8.52		1413		
	3	8.83		1495		
BRA-SBR compound modified asphalt mixture	1	9.98	9.49	2675	2692.7	4014.6

**Table 23:** Brookfield rotary viscosity of BRA-SBR compound modified asphalt (Pa·s).

T/°C	Amount of SBR (%)						Neat Asphalt
	0	2	4	5	6	8	
135	0.638	0.625	0.617	0.591	0.562	0.584	0.601
145	0.385	0.357	0.339	0.318	0.297	0.284	0.320
165	0.169	0.143	0.135	0.126	0.117	0.106	0.130
175	0.119	0.112	0.106	0.097	0.085	0.092	0.102
